# Surgical outcomes and risk factors for poor outcomes in patients with cervical spine metastasis: a prospective study

**DOI:** 10.1186/s13018-021-02562-8

**Published:** 2021-07-03

**Authors:** Yutaro Kanda, Kenichiro Kakutani, Yoshitada Sakai, Zhongying Zhang, Takashi Yurube, Shingo Miyazaki, Yuji Kakiuchi, Yoshiki Takeoka, Ryu Tsujimoto, Kunihiko Miyazaki, Hiroki Ohnishi, Yuichi Hoshino, Toru Takada, Ryosuke Kuroda

**Affiliations:** 1 Department of Orthopaedic Surgery, Kobe University Graduate School of Medicine, 7-5-1 Kusunoki-cho, Chuo-ku, Kobe, 650-0017 Japan; 2 Division of Rehabilitation Medicine, Kobe University Graduate School of Medicine, 7-5-1 Kusunoki-cho, Chuo-ku, Kobe, 650-0017 Japan; 3 Department of Orthopaedic Surgery, Kobe Hokuto Hospital, 10-3, Umekidani, Shimotanigami, Yamada-cho, Kita-ku, Kobe, 651-1243 Japan

**Keywords:** Cervical spine metastasis, Quality of life, Cervicothoracic junction, Palliative surgery, Performance status

## Abstract

**Background:**

Few studies have addressed the impact of palliative surgery for cervical spine metastasis on patients’ performance status (PS) and quality of life (QOL). We investigated the surgical outcomes of patients with cervical spine metastasis and the risk factors for a poor outcome with a focus on the PS and QOL.

**Methods:**

We prospectively analyzed patients with cervical spine metastasis who underwent palliative surgery from 2013 to 2018. The Eastern Cooperative Oncology Group PS (ECOGPS) and EuroQol 5-Dimension (EQ5D) score were assessed at study enrollment and 1, 3, and 6 months postoperatively. Neurological function was evaluated with Frankel grading. Univariate and multivariate analyses were performed to identify the risk factors for a poor surgical outcome, defined as no improvement or deterioration after improvement of the ECOGPS or EQ5D score within 3 months.

**Results:**

Forty-six patients (mean age, 67.5 ± 11.7 years) were enrolled. Twelve postoperative complications occurred in 11 (23.9%) patients. The median ECOGPS improved from PS3 at study enrolment to PS2 at 1 month and PS1 at 3 and 6 months postoperatively. The mean EQ5D score improved from 0.085 ± 0.487 at study enrolment to 0.658 ± 0.356 at 1 month and 0.753 ± 0.312 at 3 months. A poor outcome was observed in 18 (39.1%) patients. The univariate analysis showed that variables with a P value of < 0.10 were sex (male), the revised Tokuhashi score, the new Katagiri score, the level of the main lesion, and the Frankel grade at baseline. The multivariate analysis identified the level of the main lesion (cervicothoracic junction) as the significant risk factor (odds ratio, 5.00; *P* = 0.025).

**Conclusions:**

Palliative surgery for cervical spine metastasis improved the PS and QOL, but a cervicothoracic junction lesion could be a risk factor for a poor outcome.

## Background

With recent progress in cancer treatments, patients’ clinical courses have been prolonged and often accompanied by morbidity due to bone metastases. As a result, the incidence and prevalence of metastasis to the spine, which is the most common site of bone metastasis, have been increasingly rising [1]. The cervical spine is involved less frequently than the thoracic and lumbar spine, accounting for 2 to 10% of patients with spine metastasis [2]. Despite its rarity, cervical spine metastasis can be associated with tetraplegia, intractable pain, and respiratory failure, severely deteriorating the patient’s performance status (PS) and quality of life (QOL) [2–4]. It has recently become important to improve or maintain patients’ PS and QOL until the terminal phase, even in patients with stage IV cancer who develop spine metastasis [5–7]. Thus, the management of cervical spine metastasis is essential for cancer therapy.

The current treatment options for spine metastasis are surgery, radiotherapy, chemotherapy, hormonal therapy, molecular targeted drugs, and bone-modifying agents. Radiotherapy has been established as the first-choice treatment option because of its efficacy and safety. Specifically, local pain is successfully suppressed in 50 to 80% of patients with few adverse effects [8, 9]. However, treatment by radiotherapy alone has not been established for symptomatic spinal metastasis (SSM), including neurological deficits or spinal instability. Consequently, palliative surgery with decompression and stabilization is recommended for patients with SSM because of its good clinical results in terms of physical activity, pain, and neurological function [5, 10–12]. Spine surgery for SSM can also improve and maintain the PS and QOL [5, 6]. However, few publications have addressed the surgical outcome of cervical spine metastasis [3, 4, 13–16]. Furthermore, these reports focused only on objective indicators such as ambulatory function and neurological status [3, 13–15]. Thus, the effects of palliative surgery for cervical SSM on the PS and QOL have not been fully investigated. We designed a prospective cohort study of surgical outcomes of cervical SSM and analyzed the risk factors for a poor surgical outcome, with a focus on the PS and QOL.

## Methods

### Patients and surgical procedure

Fifty consecutive patients with surgical indications for cervical SSM from January 2013 to December 2018 in our institution were prospectively enrolled. Metastasis was diagnosed by plain radiography, computed tomography, magnetic resonance imaging (MRI), bone scintigraphy, position emission tomography, and histological evaluation of needle biopsy samples. As the primary site, patients with no history of malignancy whose site of primary malignancy was diagnosed after the identification of spinal metastasis were categorized into “unknown.” The surgical indications were progressive neurological deficits, remarkable spinal instability (Spinal Instability Neoplastic Score [17] of ≥ 13), and intractable pain resistant to conservative care, including the use of opioids. The contraindication for surgery was impaired consciousness due to cerebral metastasis. All surgeries involved single-stage posterior decompression with partial removal of the tumor to a feasible extent from the posterolateral aspect and posterior stabilization with fixation using lateral mass screws for C1 and C3–6 and pedicle screws for C2, C7, and the thoracic spine. Neither an anterior approach nor a combined approach was used. All collars were removed postoperatively. All patients underwent radiotherapy before or after surgery. If indicated, chemotherapy was performed by the oncologist.

The postoperative survival duration was defined as the time from the date of surgery to the latest follow-up examination or death. At the start of the study (baseline), we investigated the age, sex, and the primary site as preoperative factors. The new Katagiri score [18] and the revised Tokuhashi score [12] were used to predict the prognosis and assess the severity of spinal metastases. Frankel grading [19] was used to evaluate neurological function. The level of the main lesion (upper cervical spine, C1–2; middle cervical spine, C3–6; or cervicothoracic junction; C7–T1), Spinal Instability Neoplastic Score [17], and epidural spinal cord compression grade [20] were used to assess the type of spine metastasis. The Eastern Cooperative Oncology Group PS (ECOGPS) [21] and EuroQol 5-Dimension (EQ5D) score [22] were used to evaluate the PS and QOL, respectively. The operative time, blood loss, number of fixed vertebrae, and postoperative complications were investigated as surgery-related factors. The Clavien–Dindo classification was used to evaluate the severity of postoperative complications, and severe complications were defined as grade ≥ III [23]. Clinical follow-up examinations were routinely performed at 1, 3, and 6 months postoperatively. Improvement and deterioration of the PS and QOL were defined as a ≥ 1-level change in the PS and a ≥ 10% change in the EQ5D score, respectively. Based on these definitions, the improvement rate, deterioration rate, and re-deterioration rate within 6 months were calculated. In addition, a poor surgical outcome was defined as no improvement or deterioration after improvement of the ECOGPS or EQ5D score within 3 months. Patients who were alive and could not consult our department were contacted by telephone to obtain the latest follow-up information. For patients who died, we obtained information from the patient’s family or institution of transfer.

### Statistical analysis

All statistical analyses were performed using SPSS 23.0 (IBM Corp., Armonk, NY, USA) with statistical significance set at a *P* value of < 0.05. Values are expressed as mean ± standard deviation or median and interquartile range. The overall survival rate was calculated by the Kaplan–Meier method. To identify the risk factors for a poor surgical outcome, the Mann–Whitney U test was used for continuous variables, and the chi-square test or Fisher’s exact test was used for categorical variables. All variables with a *P* value of < 0.10 in the univariate analysis were considered potential risk factors and entered into the multivariate backward logistic regression analysis.

## Results

### Patient characteristics and surgical results

**Fig. 1:**
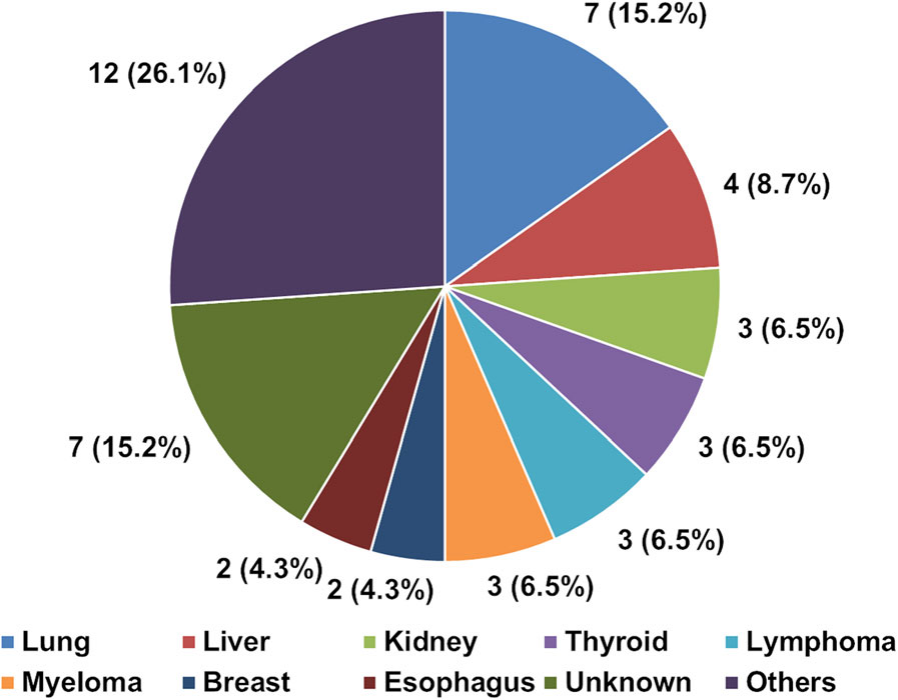
Primary tumor type at surgery

Three patients were excluded because of treatment contraindications. One patient who was initially suspected to have metastases but instead had a primary vertebral osteosarcoma was also excluded. Consequently, 46 patients were enrolled. No patients were lost to follow-up. Preoperative factors and surgery-related factors are shown in Table 1. The primary sites are shown in Fig. 1. Radiotherapy was performed in 15 (32.6%) patients before surgery, whereas postoperative radiotherapy was performed in 31 (67.4%) patients. Long-course (> 10 fractions) external beam radiation therapy (EBRT) was performed in 36 (78.3%) patients, whereas 8-Gy/single fraction EBRT was performed in one (2.2%) patient. Intensity-modulated radiation therapy (IMRT) was performed in nine (19.6%) patients. Chemotherapy was performed in 17 (37.0%) patients before surgery. Postoperative chemotherapy was performed in 21 (47.5%) patients. Twelve postoperative complications occurred in 11 (23.9%) patients. The most common postoperative complications were wound disorders: radiation dermatitis occurred in three patients (6.5%), and surgical site infection occurred in two patients (4.3%). Four (8.7%) patients developed severe complications (reoperation due to radiation dermatitis and infection, acute myocardial infarction, and hydrocephalus). No patients developed implant failure.

**Table 1:** Demographics and clinical characteristics of patients at the surgery

Characteristics	
Age, years	67.5 ± 11.7
Male sex	30 (65.2)
Revised Tokuhashi score, points	6.6 ± 2.5
New Katagiri score, points	4.7 ± 2.0
SINS, points	11.4 ± 2.7
ESCC grade	
Grades 0 and 1	14 (30.4)
Grades 2 and 3	32 (69.6)
Level of the main lesion	
Upper cervical spine	9 (19.6)
Middle cervical spine	18 (39.1)
Cervicothoracic junction	19 (41.3)
ECOGPS	
PS 1	2 (4.3)
PS 2	10 (21.7)
PS 3	22 (47.8)
PS 4	12 (26.1)
EQ5D, points	0.085 ± 0.487
Frankel grade	
Grades A and B	1 (2.2)
Grades C and D	29 (63.0)
Grade E	16 (34.8)
Operative time, min	188.8 ± 71.2
Blood loss, g	276.8 ± 301.1
Number of fixed vertebrae	5.8 ± 2.8

Data are presented as *n* (%) or mean ± standard deviation

*SINS*, Spinal Instability Neoplastic Score; *ESCC*, Epidural Spinal Cord Compression; *ECOGPS*, Eastern Cooperative Oncology Group Performance Status; *EQ5D*, EuroQol 5-Dimension

### Survival rate

**Fig. 2:**
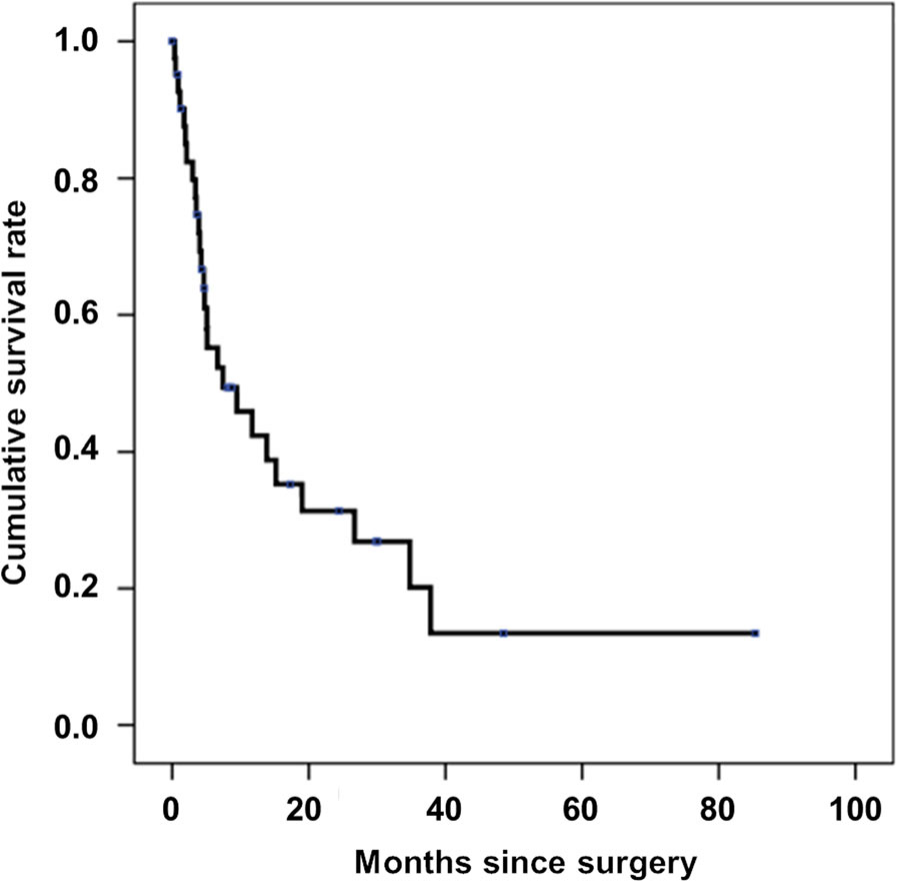
Kaplan–Meier survival curve

One patient died of primary cancer 1 month postoperatively. The number of surviving patients was 45 (survival rate, 97.8%) at 1 month, 32 (69.6%) at 3 months, and 19 (41.3%) at 6 months. The median survival time was 7.4 months (95% confidence interval, 1.9–17.7 months) (Fig. 2). Finally, 37 (80.4%) patients returned home, and 9 (19.6%) patients were transferred to other hospitals, including hospice care facilities.

### Clinical outcomes

**Fig. 3:**
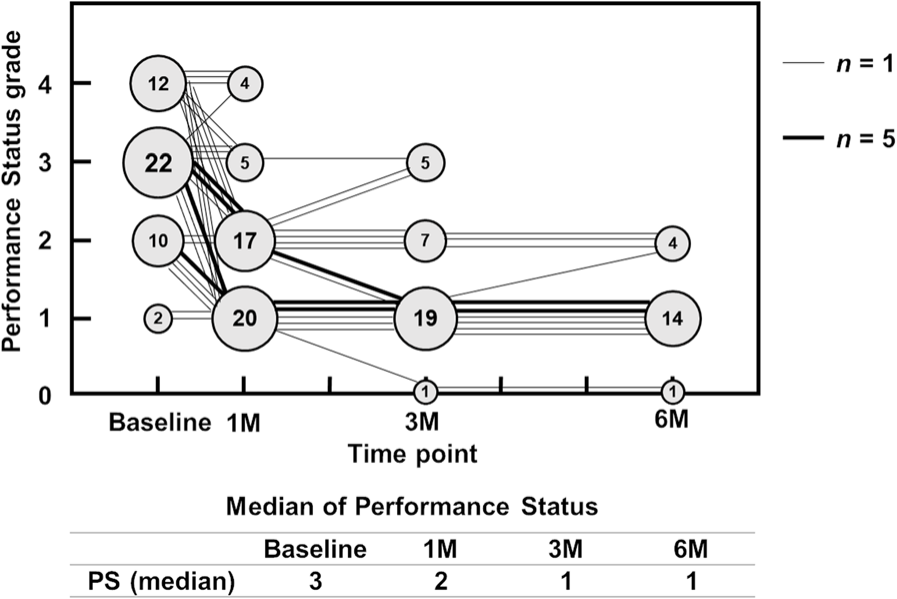
PS preoperatively and at 1, 3, and 6 months postoperatively. The figures in the circles indicate the numbers of patients. The line connecting the circles shows the transition of an individual patient. The thick line represents five patients. PS, performance status

Of all 46 patients, 34 (73.9%) had a PS of ≥ 3 at the start of the study, and 37 (80.4%) had a PS of ≤ 2 at 1 month postoperatively. Consequently, the median PS improved from PS3 at the start of the study to PS2 at 1 month postoperatively. It further improved to PS1 at 3 months postoperatively, and the acquired PS level was maintained until 6 months (Fig. 3).

**Fig. 4:**
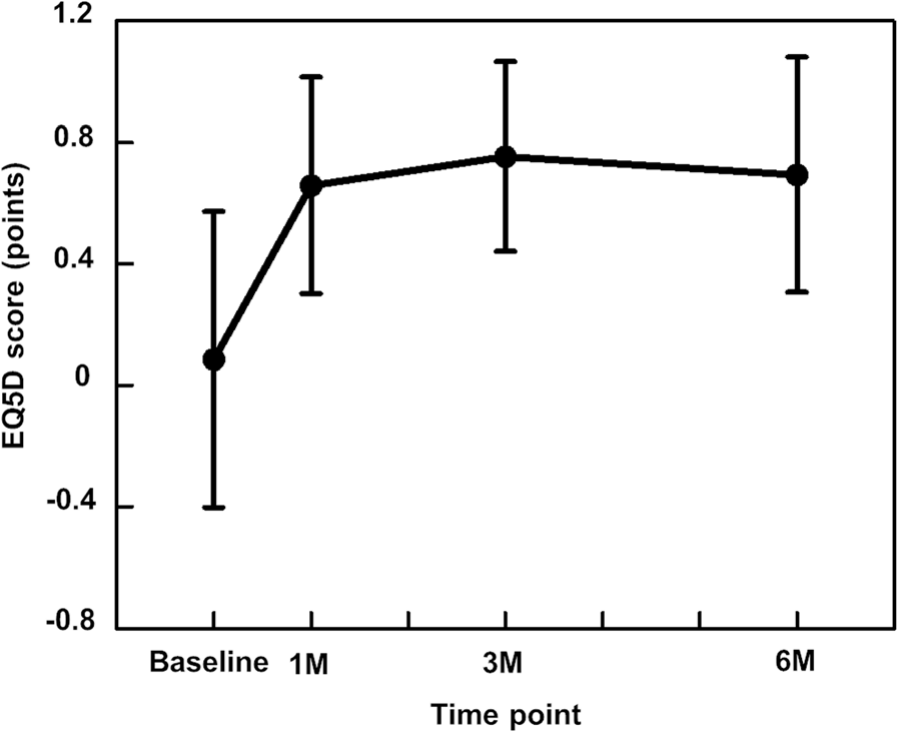
EQ5D score preoperatively and at 1, 3, and 6 months postoperatively. EQ5D, EuroQol 5-Dimension

The mean EQ5D score at the start of the study was 0.085 ± 0.487. It improved to 0.658 ± 0.356 at 1 month postoperatively and further improved to 0.753 ± 0.312 at 3 months postoperatively. The EQ5D score then slightly deteriorated to 0.693 ± 0.387 at 6 months postoperatively (Fig. 4).

**Fig. 5:**
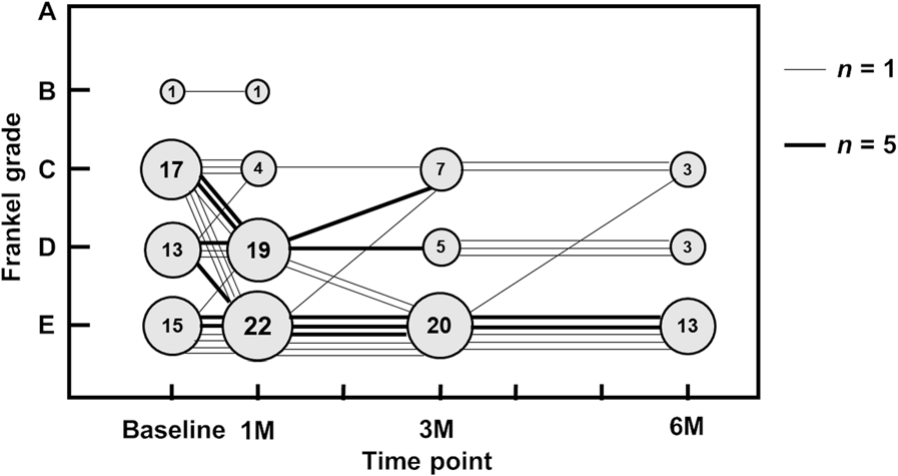
Frankel grading preoperatively and at 1, 3, and 6 months postoperatively. The figures in the circles indicate the numbers of patients. The line connecting the circles shows the transition of an individual patient. The thick line represents five patients

Chronological changes in the Frankel grade during observation are shown in Fig. 5. Almost all neurologically normal patients (Frankel grade E) retained their neurological status. Among 30 patients with Frankel grades C and D at the baseline, 14 patients had a ≥ 1-level improvement and 10 patients had no improvement at 6 months after surgery.

**Fig. 6:**
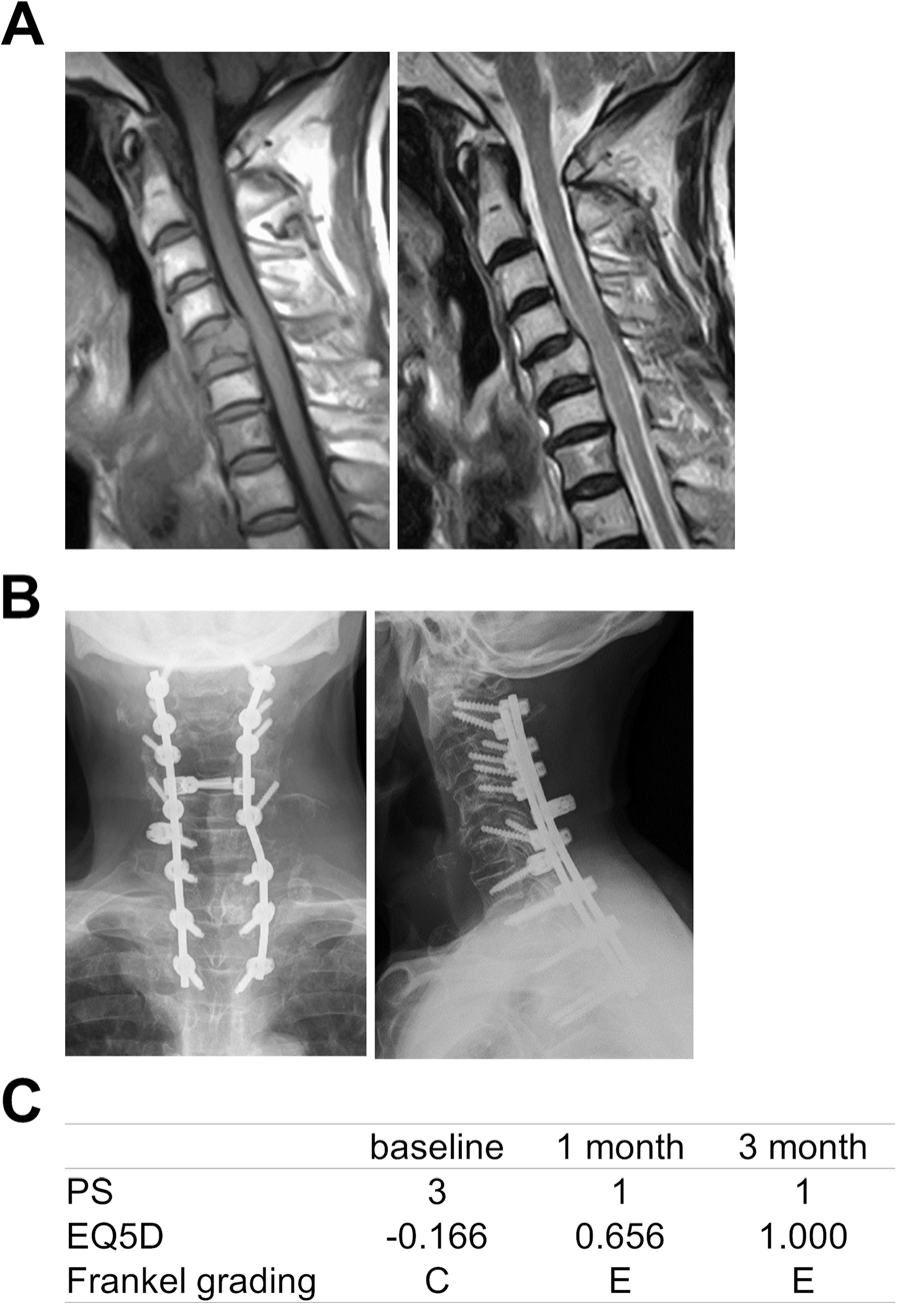
Images and clinical course of the 67-year-old man with metastatic thyroid cancer to the C5 vertebra. **A** Preoperative MRI: sagittal T1-weighed enhanced image at the left side and sagittal T2-weighed enhanced image at the right side. **B** Postoperative radiographs: posteroanterior image at the left side and lateral image at the right side. **C** PS, EQ5D, and Frankel grading preoperatively and at 1 and 3 months postoperatively. MRI, magnetic resonance imaging; PS, performance status; EQ5D, EuroQol 5-Dimension

**Fig. 7:**
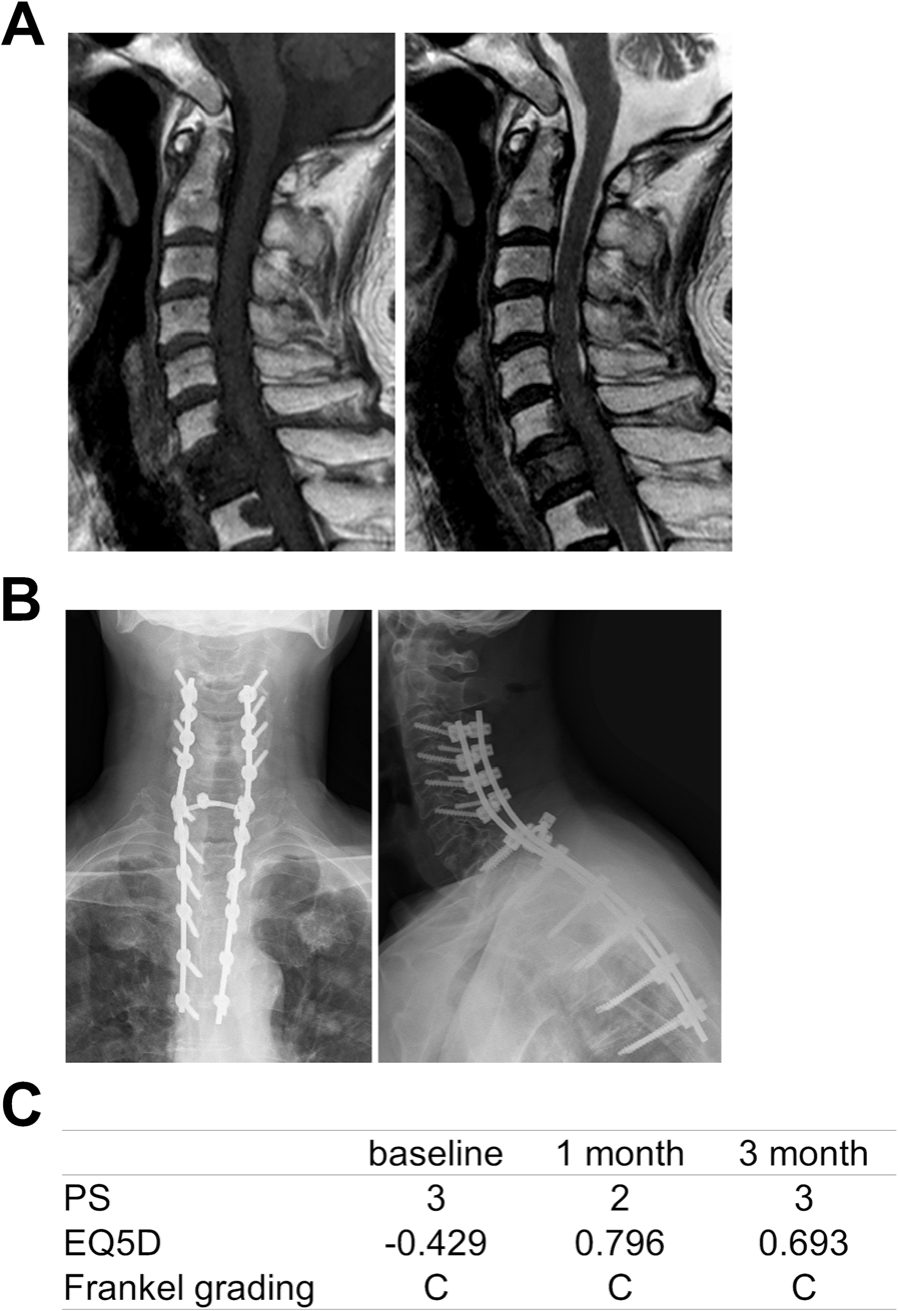
Images and clinical course of the 69-year-old man with metastatic hepatocellular carcinoma to the C7 vertebra. **A** Preoperative MRI: sagittal T1-weighed enhanced image at the left side and sagittal T2-weighed enhanced image at the right side. **B** Postoperative radiographs: posteroanterior image at the left side and lateral image at the right side. **C** PS, EQ5D, and Frankel grading preoperatively and at 1 and 3 months postoperatively. MRI, magnetic resonance imaging; PS, performance status; EQ5D, EuroQol 5-Dimension

Consequently, > 80% of patients showed an improvement in the PS and EQ5D score. Images and clinical course of a typical patient who had an excellent outcome after surgery are shown in Fig. 6. However, some patients’ health state and neurological status deteriorated after the initial improvement. Images and clinical course of a typical patient who had a poor outcome after surgery are shown in Fig. 7. Re-deterioration of the PS, EQ5D score, and Frankel grade was observed in 7 of 38 (18.4%) patients, 8 of 41 (19.5%) patients, and 7 of 21 (33.3%) patients, respectively (Table 2).

**Table 2:** Individual chronological changes in PS, QOL, and neurological status

	*n* (%)
PS	
Improvement	38 (82.6)
No change	6 (13.0)
Deterioration	2 (4.3)
Re-deterioration	7 (18.4)
QOL	
Improvement	41 (89.1)
No change	3 (6.5)
Deterioration	2 (4.3)
Re-deterioration	8 (19.5)
Neurological status	
Improvement	21 (45.7)
No change	22 (47.8)
Deterioration	3 (6.5)
Re-deterioration	7 (33.3)

*PS*, performance status; *QOL*, quality of life

### Analysis of risk factors for poor surgical outcome

A poor surgical outcome occurred in 18 (39.1%) patients. No improvement or deterioration after improvement of the ECOGPS and EQ5D score within 3 months was observed in 15 and 13 patients, respectively. The univariate analysis showed that variables with a *P* value of < 0.10 were sex (male), the revised Tokuhashi score, the new Katagiri score, the level of the main lesion, and the Frankel grade at baseline (Table 3). The multivariate analysis identified the level of the main lesion as the only significant risk factor (odds ratio, 5.00; *P* = 0.025) (Table 4). The chi-square test showed that the presence of the main lesion at the cervicothoracic junction was a significant risk factor for a poor outcome of surgery for SSM (*P* = 0.006).

**Table 3:** Univariate analysis of risk factors for poor outcome

Factors	Good outcome (*n* = 28)	Poor outcome (*n* = 18)	*P*
Age			0.639
≥ 65 years	19 (41.3)	11 (23.9)	
< 65 years	9 (19.6)	7 (15.2)	
Sex			0.039*
Male	13 (28.2)	3 (6.5)	
Female	15 (32.6)	15 (32.6)	
Revised Tokuhashi score, points	7.4 ± 2.7	5.2 ± 1.4	0.002*
New Katagiri score, points	4.1 ± 2.2	5.6 ± 1.2	0.013*
SINS, points	11.7 ± 2.8	10.9 ± 2.4	0.335
Level of the main lesion			0.006*
Upper cervical spine (C1–2)	8 (17.3)	1 (2.2)	
Middle cervical spine (C3–6)	13 (28.3)	5 (10.9)	
Cervicothoracic junction (C7, T1)	7 (15.2)	12 (26.1)	
ESCC grade at baseline			0.732
Grade ≥ 2	20 (43.5)	12 (26.1)	
Grade ≤ 1	8 (17.4)	6 (13.0)	
ECOGPS at baseline			0.953
PS ≥ 3	20 (43.5)	13 (28.3)	
PS ≤ 2	8 (17.4)	5 (10.9)	
Frankel grade at baseline			0.016*
Grades A and B	0 (0.0)	1 (2.2)	
Grades C and D	14 (30.4)	15 (32.6)	
Grade E	14 (30.4)	2 (4.3)	
Preoperative chemotherapy			0.142
Yes	8 (17.4)	9 (19.6)	
No	20 (43.5)	9 (19.6)	
Preoperative radiotherapy			0.466
Yes	8 (17.4)	7 (15.2)	
No	20 (43.5)	11 (23.9)	

Data are presented as *n* (%) or mean ± standard deviation

*SINS*, Spinal Instability Neoplastic Score; *ESCC*, Epidural Spinal Cord Compression; *ECOGPS*, Eastern Cooperative Oncology Group Performance Status

*Statistically significant at *P* < 0.100

**Table 4:** Multivariate analysis of risk factors for poor outcome

	Odds ratio	95% confidence interval	*P*
Sex (male)	2.83	0.40–19.98	0.296
Revised Tokuhashi score	0.67	0.43–1.04	0.076
New Katagiri score	1.13	0.66–1.91	0.660
Level of the main lesion	5.00	1.23–20.32	0.025*
Frankel grade at baseline	0.54	0.08–3.85	0.537

*Statistically significant at *P* < 0.05

## Discussion

Multiple studies have shown excellent clinical outcomes of palliative surgery for patients with spinal metastasis in terms of neurological function and pain control [5, 10–12]. Surgery for patients with spine metastasis has also recently been highlighted in terms of the PS and QOL [5–7], improvements in which should be set as a treatment goal. With respect to cervical spine metastasis, some studies have shown the effectiveness of surgery in terms of neurological recovery and pain control [3, 4, 13–16]. However, few studies have focused on the PS and QOL [4, 16, 24]. Therefore, to clarify the impact of palliative surgery for cervical spine metastasis on the PS and QOL, we prospectively investigated the ECOGPS and EQ5D score in addition to objective indicators.

In a retrospective study of 34 patients with cervicothoracic junctional spine metastasis (C7–T2) [24], the Karnofsky PS was maintained or improved in 32 (94.1%) patients. In addition, a retrospective study of 57 patients with cervical spine metastasis showed that the mean Karnofsky PS improved from 54.5 preoperatively to 64.7 on postoperative day 14 [4]. Other reports of the surgical outcomes of cervical spine metastasis did not mention the PS [3, 13–15]. The current study showed a sustainable improvement in the median ECOGPS. Furthermore, > 80% of patients showed an improvement in the PS, suggesting the effectiveness of palliative surgery for cervical spine metastasis in terms of the PS.

QOL has recently drawn increasing attention as an indicator of the therapeutic effect in patients with cancer. Various scoring systems have been used to assess QOL, including the European Organization for Research and Treatment of Cancer QOL Core Questionnaire 30 (EORTC QLQ-30), the Functional Assessment of Cancer Therapy-General (FACT-G), the Short Form-36 (SF-36), and the EQ5D. Because cervical SSM can cause paraplegia and directly affect patients’ QOL, assessment of QOL is essential for accurate judgment of the natural course and therapeutic effect of cervical SSM. However, only one article to date has assessed the effectiveness of surgery for patients with cervical spine metastasis in terms of QOL [16]. A prospective series of 26 patients with cervical or cervicothoracic spine metastasis showed an improvement in the pain, global health, and cognitive functioning domains of the EORTC QLQ-30 [16]. Notably, however, cervical spine metastasis is regarded to have a poorer prognosis and worse neurological recovery than thoracic and lumbar spine metastasis [14]. There is concern about the effectiveness of palliative surgery for cervical spine metastasis with respect to improvement in QOL. In the current study, the mean EQ5D score improved from nearly 0 at study enrolment to around 0.7 throughout the entire postoperative period. This result indicates that the patients’ QOL was almost equivalent to death before surgery and greatly improved to a satisfactory level after surgery. Although surgery for cervical SSM might be more technically difficult than that for thoracic and lumbar SSM, it appears to be valuable in terms of the PS and QOL.

We also analyzed the result of this study from a critical viewpoint. Based on another definition (a ≥ 1-level change in the PS or a ≥ 10% change in the EQ5D score in each patient), some patients experienced re-deterioration of their PS and QOL. We observed a poor outcome, defined as no improvement or re-deterioration of the PS or QOL, in more than one-third of patients. This fact should not be overlooked and is useful for predicting a poor outcome and selecting more appropriate surgical indications. Our univariate analysis showed that the new Katagiri score, revised Tokuhashi score, and preoperative neurological status were risk factors for a poor outcome. These results are supported by prior reports describing poor survival [12, 18] and ambulatory function [25]. However, these factors were excluded by the multivariate analysis in the current study. A multivariate analysis involving 19 patients with cervical spine metastasis identified the type of primary tumor, preoperative ambulatory status, and presence of extra-spinal bone metastasis as risk factors affecting postoperative survival [25]. Our multivariate analysis identified a cervicothoracic junction lesion as a risk factor for a poor outcome. Interestingly, a retrospective analysis of 81 patients with spine metastasis, including both the thoracic and lumbar spine, showed that metastasis at the upper thoracic spine or cervicothoracic junction is a risk factor for a poor neurological functional outcome [26]. Considering that distal cervical spondylotic amyotrophy is also likely to result in a poor neurological outcome [27], the anatomical characteristics of the cervicothoracic junction may affect these results. In addition, surgical site infection most commonly occurred at the cervicothoracic junction, which is consistent with a prior report [28]. It is possible that patients with wound complications requiring long-term negative-pressure wound therapy or administration of antibiotics are less likely to undergo adequate rehabilitation.

This study has three main limitations. First, the follow-up period varied among patients and was relatively short. However, this was inevitable because of the characteristics of patients with stage IV cancer. Actually, the overall median postoperative survival time was < 8 months. Second, the only surgical approach used in the current study was the posterior approach. Available approaches include the anterior, posterior, and combined approaches. The anterior approach allows for direct debulking of the volume of the metastatic lesion, which is often located in the vertebral body; however, its disadvantages include excessive bleeding and difficulty in anterior reconstruction when multiple vertebrae are affected. Posterior approaches with decompressive laminectomy followed by stabilization have recently become the most common surgical procedures for cervical spine metastasis because of the ease and stability of posterior instrumentation. Only the posterior approach may be preferable in terms of procedure homogeneity, whereas the anterior or combined approach may be desirable in terms of anterior reconstruction and debulking the volume of the metastatic lesion. Insufficiency of anterior decompression may affect the results of the poor outcome in patients with cervicothoracic junctional metastasis. Third, the sample size was relatively small. Further study is needed.

## Conclusions

Although the effects of palliative surgery for cervical spine metastasis on the PS and QOL have not been fully investigated, the current study suggests that surgery should be recommended because of its potential to improve the PS and QOL. However, a cervicothoracic junction lesion could be a risk factor for no improvement or re-deterioration after improvement of the PS or QOL.

## Data Availability

The datasets analyzed during the current study are available from the corresponding author on reasonable request.

## Abbreviations

PS: Performance status
QOL: Quality of life
ECOGPS: Eastern Cooperative Oncology Group Performance Status
EQ5D: EuroQol 5-Dimension
SSM: Symptomatic spinal metastasis
EORTC QLQ-30: European Organization for Research and Treatment of Cancer QOL Core Questionnaire 30
FACT-G: Functional Assessment of Cancer Therapy-General
SF-36: Short Form-36
